# The Utility of Intravenous Methylprednisolone as an Adjunct Treatment for Drug-Resistant Amiodarone-Induced Thyrotoxicosis

**DOI:** 10.3390/jcm13020324

**Published:** 2024-01-06

**Authors:** Krzysztof Cezary Lewandowski, Joanna Kawalec, Michał Kusiński, Katarzyna Dąbrowska, Aleksandra Ewa Matusiak, Iga Dudek, Andrzej Lewiński

**Affiliations:** 1 Department of Endocrinology and Metabolic Diseases, Medical University of Lodz, 93-338 Lodz, Poland; krzysztof.lewandowski@umed.lodz.pl (K.C.L.); aleksandra.matusiak@umed.lodz.pl (A.E.M.); 2 Department of Endocrinology and Metabolic Diseases, Polish Mother’s Memorial Hospital—Research Institute, 93-338 Lodz, Poland; joanna.kawalec@iczmp.edu.pl (J.K.); katarzyna.dabrowska@iczmp.edu.pl (K.D.); iga.dudek@iczmp.edu.pl (I.D.); 3 Department of Endocrinological, General and Vascular Surgery, Medical University of Lodz, 93-338 Lodz, Poland; michal.kusinski@umed.lodz.pl; 4 Department of Endocrine, General and Oncological Surgery, Mikolaj Kopernik District Hospital, 91-513 Lodz, Poland; 5 Department of Paediatric Endocrinology, Medical University of Lodz, 93-338 Lodz, Poland

**Keywords:** steroids, pulse methylprednisolone, goitre, amiodarone-induced thyrotoxicosis

## Abstract

Background: Amiodarone-induced thyrotoxicosis (AIT) may pose treatment challenges. We present a series of patients in which we achieved the normalisation of free T3 (FT3) using intravenous methylprednisolone (ivMP) in AIT refractory to thiamazole and oral prednisone. Namely, in three males (aged 56, 50 and 64, all with a history of AF and/or a low ejection fraction), an addition of ivMP resulted in the normalisation of FT3, which allowed successful thyroidectomy. In another case of a 65-year-old man, we initially succeeded in the normalisation of FT3 using ivMP from FT4 > 7.77 ng/dL (0.93–1.7) to 2.41 ng/dL and in that of FT3 from 14.95 pg/mL (2–4.4) to 2.05 pg/mL), but four weeks after stopping ivMP, despite the continuation of thiamazole and prednisone, there was rebound thyrotoxicosis: FT4 > 7.77 ng/dL and FT3—5.46 pg/mL. Intravenous MP was restated leading to a decline in FT4 to 2.51 ng/dL and in FT3 to 1.92 pg/mL, thus allowing a successful thyroidectomy. Finally, in a 78-year-old man with AF, goitre, and AIT resistant to thiamazole, prednisone and lithium carbonate, we obtained a reduction in FT4 to 1.51 ng/dL and in FT3 to 3.17 pg/mL after seven pulses of ivMP. Oral prednisone was gradually reduced and successfully stopped about six months later. He remained on low-dose thiamazole (5 mg od). Conclusions: Pulse ivMP in addition to oral steroids may be a useful adjunct therapy either for the preparation of a thyroidectomy or as a treatment modality in drug-resistant AIT. Though a total cure is possible, there is a danger of a rebound worsening of thyrotoxicosis after premature discontinuation of ivMP.

## 1. Introduction

Amiodarone-induced thyrotoxicosis (AIT) is typically divided into type 1, a form of iodine-induced hyperthyroidism, occurring in patients with nodular goiters or latent Graves’ disease, and type 2, a drug-induced destructive thyroiditis. However, mixed/indefinite forms exist and indeed predominate in more serious cases, while distinction might be extremely difficult on clinical grounds [1]. Type 1 thyrotoxicosis is typically treated with thionamides, while type 2 is treated with glucocorticoids. Several other treatment options were tried in resistant cases, including sodium perchlorate [2], lithium carbonate [3], cholestyramine [4] or a combination of several treatment modalities, e.g., thionamides, oral steroids, lithium and cholestyramine [5]. Though general guidelines exist [1,2,6], the management of individual cases may be complex, with resistance to treatment, as well as rebound increases in thyroid hormone concentrations despite therapy.

We present a series of cases of drug-resistant AIT that required eventual thyroidectomy, in which we achieved the normalisation (or near normalisation) of free T3 (FT3) levels using pulse methylprednisolone (MP) treatment prior to surgery, as well as the case of an elderly patient, in whom we managed to avoid thyroidectomy due to treatment with a combination of oral prednisone and pulsed intravenous MP (ivMP).

## 2. Case Presentations

### 2.1. Case I

**Figure 1:**
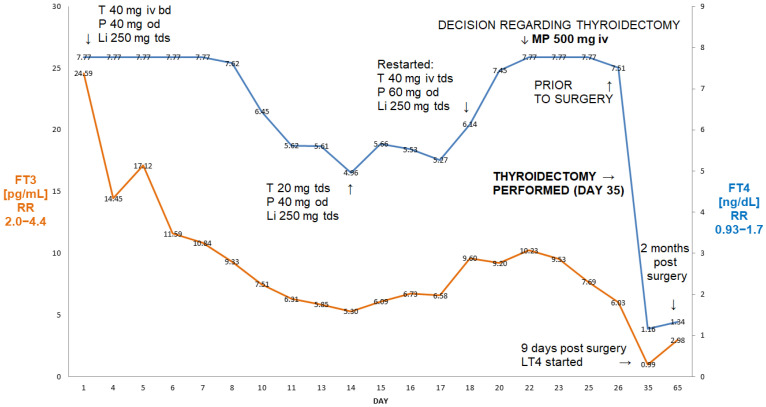
Summary of concentrations of free T4 and free T3 of a 56-year-old male patient with AIT (Case I). After initial improvement on thiamazole and oral glucocorticoids, there was a rebound deterioration despite medical treatment. The administration of 500 mg of ivMP resulted in a fall in free T3 from 10.23 pg/mL to 6.03 pg/mL (41% decrease in FT3) within 72 h, allowing for the better preparation of this patient for thyroidectomy. Abbreviations: iv—intravenous; T—thiamazole; P—prednisone; Li—lithium carbonicum; od—once a day; bd—twice a day; tds—three times a day; LT4—levothyroxine; FT4—free T4; FT3—free T3; RR—reference range; MP—methylprednisolone. Arrows indicate either change of medication, or timing of MP administration.

A 56-year-old man (BMI 29.7 kg/m^2^) with a history of atrial flutter/fibrillation and episodes of rapid AF~200/minute, after unsuccessful ablation therapy, presented with severe AIT and was unresponsive to high-dose oral thiamazole (40 mg/day) and oral prednisone. Thiamazole had been used for three months and prednisone had been used for about six weeks prior to his admission to the hospital. Amiodarone was stopped by a cardiologist about six weeks prior to his admission. On admission, his FT3 was 24.6 pg/mL (reference range 2.0–4.4), free T4 (FT4) was >7.77 ng/dL (reference range 0.93–1.7) and TSH was <0.005 µIU/mL (reference range 0.27–4.2). A thyroid ultrasound revealed a normal-sized thyroid without focal lesions and without evidence of an enhanced flow on echo-colour Doppler. An autoimmune screen for anti-thyroid peroxidase (aTPO), anti-thyroglobulin (aTG) and anti-TSH-receptor antibodies was negative. The patient’s results and course of treatment are presented in Figure 1.

He initially responded to high-dose intravenous thiamazole (Thiamazol 40 mg inject, Henning^®^, Sanofi, France), 40 mg tds, lithium carbonate, 250 mg tds and oral prednisone (Encorton^®^, Adamed Pharma, Poland) and 40 mg od, i.e., after 11 days, his FT3 was 5.4 pg/mL, FT4—4.96 ng/dL, but after the change to oral thiamazole (20 mg tds), there was a rebound increase in FT3 (to 9.6 pg/mL) and FT4 (to 6.14 ng/dL), followed by episodes of rapid atrial fibrillation, despite an increase in oral prednisone to 60 mg od and thiamazole to 40 mg tds from day 18 (Figure 1). This prompted his admission to the Department of Cardiology, where he was treated with high-dose intravenous metoprolol, diuretics and spironolactone in view of developing oedema. The re-administration of intravenous thiamazole prevented an increase in FT3, but there was a further increase in FT4 (>7.77 ng/dL)—see Figure 1. Despite the use of high-dose beta-blockers (bisoprolol 20 mg/day), he remained persistently tachycardic, with about 120 beats per minute. On the 24th day of admission, he was therefore referred for an emergency thyroidectomy. The administration of 500 mg of ivMP (in addition to oral prednisone) within 72 h resulted in a 41% decrease in FT3 from 10.23 pg/mL to 6.03 pg/mL (2.0–4.4), i.e., finally only 37% above the upper reference range. Following the administration of two units of fresh frozen plasma (in order to enhance further thyroid hormone binding), he underwent successful total thyroidectomy on day 27 post-admission. Nine days after the thyroidectomy, his TSH was still suppressed at 0.005 µIU/mL, but his FT3 and FT4 were low (FT3—0.99 pg/mL, FT4—1.16 ng/dL, respectively), and levothyroxine was stated. He remains currently euthyroid on levothyroxine 150 µg od. His planned cardioversion was cancelled as he reverted spontaneously to a sinus rhythm about two months post-surgery.

### 2.2. Case II

A 50-year-old man (BMI 26.87 kg/m^2^), with severe heart failure due to dilated cardiomyopathy (EF = 14%), awaiting myocardial transplantation, was admitted to the Department of Endocrinology and Metabolic Diseases due to AIT, and was unresponsive to high-dose oral thiamazole (60 mg/day) that had been used for at least two months prior to admission. His history revealed that since then, he had lost about 25 kg in about four months and complained about weakness, fatigue and increased sweating. The patient had a history of atrial fibrillation treated with amiodarone for about six months prior to admission to our Department. In June 2022, he underwent an implantation of a cardioverter–defibrillator (Boston Scientific Charisma VVI, USA).

On admission to our Department, the patient was in stable condition, with clinical and biochemical features of thyrotoxicosis. Amiodarone treatment was discontinued and autoimmune thyroid disease was excluded (negative titres of aTPO, aTG and anti-TSH receptor antibodies). A thyroid ultrasound revealed a normal-sized thyroid without focal lesions and with decreased vascular flow on power Doppler examination.

**Figure 2:**
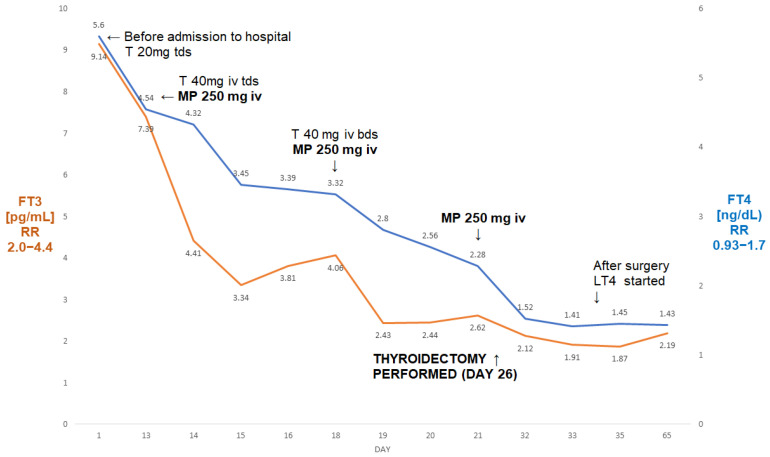
A summary of concentrations of free T4 and free T3 of a 50-year-old male patient with AIT (Case II). The administration of three pulses of 250 mg of ivMP resulted in the normalisation of free T3 prior to the thyroidectomy. Abbreviations: iv—intravenous; T—thiamazole; bd—twice a day; tds—three times a day; LT4—levothyroxine; FT4—free T4; FT3—free T3; RR—reference range; MP—methylprednisolone.

On admission, his FT3 was 7.39 pg/mL, FT4 was 4.54 ng/dL, and TSH was <0.005 µIU/mL. During admission to our department, he received treatment for thyrotoxicosis with high doses of intravenous thiamazole (120 mg/day, later reduced to 80 mg/day), together with MP iv pulses (three pulses of 250 mg intravenously each)—see Figure 2. This was followed by s normalisation of FT3 (from 7.39 pg/mL to 2.44 pg/mL), and a fall in FT4 from 4.54 ng/dL to 2.28 ng/dL.

He subsequently underwent a successful total thyroidectomy (six days post-surgery: TSH—0.07 µIU/mL, FT3—1.52 pg/mL, FT4—2.12 ng/dL). There was a period of post-operative hypotension, but this was corrected after a reduction in his antihypertensive medication. Five weeks post-surgery, he had mild hypothyroidism on levothyroxine 100 µg od (FT4—1.43 ng/dL; FT3—2.19 pg/mL; TSH—7.69 µIU/mL). Echocardiography showed some increase in the ejection fraction to 20%. To the best of our knowledge, he is still awaiting his heart transplant.

### 2.3. Case III

**Figure 3:**
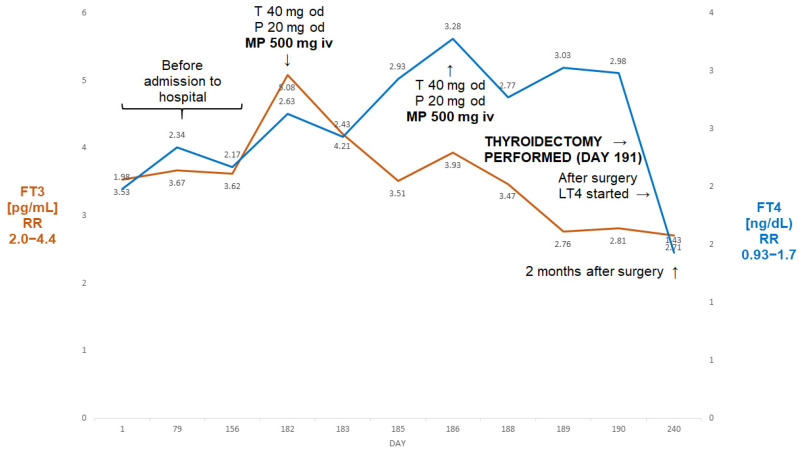
Summary of concentrations of free T4 and free T3 of 64-year-old male patient with AIT (Case II). The administration of two pulses of 500 mg of ivMP resulted in the normalisation of free T3 prior to the thyroidectomy despite the failure of prior medical treatment (thiamazole and oral prednisone). Abbreviations: iv—intravenous; T—thiamazole; P—prednisone; od—once a day; LT4—levothyroxine; FT4—free T4; FT3—free T3; RR—reference range; MP—methylprednisolone.

A 64-year-old man, BMI 21.8 kg/m^2^, was admitted to our department because of refractory AIT. At the age of 50, he had undergone mitral valve surgery with the implantation of a Carpenter-Edwards 34 mm ring. Around the age of 61, he developed paroxysmal atrial fibrillation treated with ablation therapy at the age of 62. He subsequently, however, developed non-ischaemic cardiomyopathy (ejection fraction 30%) with a history of both supraventricular and ventricular arrhythmias. A cardioverter was implanted and amiodarone treatment was started around 10 months prior to admission (TSH four months later 0.91 µIU/mL). Following a cardiological assessment, he was thought to require aortic valve surgery due to developing insufficiency. About three months later, he was, however, found to have partially suppressed TSH (0.11 µIU/mL) with FT3 within the reference range (Figure 3).

Two months later, TSH became fully suppressed (0.04 µIU/mL). Amiodarone was stopped and he was started on thiamazole 20 mg bd and oral prednisone 20 mg od. Despite undergoing about five weeks of that treatment, about a month later, his FT3 was seen to be rising (Figure 3) and he was deemed unsuitable for planned valve surgery. He was subsequently admitted to our department, where he received two pulses of ivMP (one week apart) in addition to thiamazole and oral prednisone. A thyroid ultrasound revealed a normal-sized thyroid without focal lesions, without evidence of an enhanced flow on power Doppler. The titres of all thyroid antibodies were normal. This resulted in an almost 50% fall in FT3, and the patient underwent successful a thyroidectomy. Two months later he was euthyroid on levothyroxine 100 µg od (TSH—2.12 µIU/mL; FT3—2.71 pg/mL; FT4—1.43 ng/dL).

### 2.4. Case IV

A 65-year-old man was admitted to our department because of drug-resistant AIT. He had a history of atrial fibrillation (AF), treated with amiodarone since 2019, and non-ST-elevation myocardial infarction (NSTEMI) in 2020. In 2022, he suffered another NSTEMI and was found to be thyrotoxic (TSH < 0.005 µIU/mL; FT3 22.4 pmol/L (reference range 3.1–6.8), FT4 > 100 pmol/L (reference range 12–22)). Amiodarone was stopped, but he failed to respond to thiamazole (20 mg tds administered for about two months prior to his admission).

**Figure 4:**
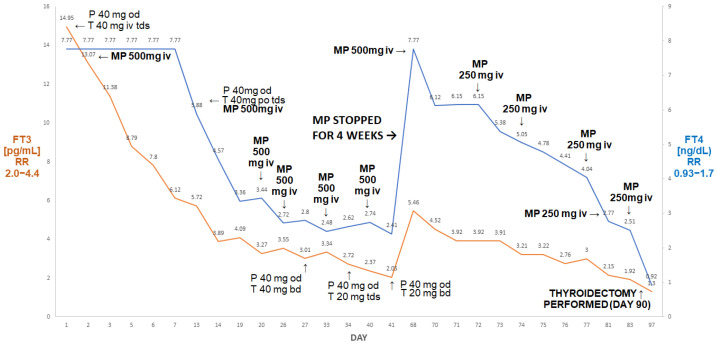
Summary of concentrations of free T4 and free T3 of the 65-year-old male patient with AIT. After initial improvement on pulse MP there was a rebound deterioration of MP withdrawal (despite the continuation of oral thiamazole and prednisone). The reinstitution of pulse MP treatment was followed by a marked fall in free T4 and free T3, resulting in the normalisation of free T3 prior to a successful thyroidectomy. Abbreviations: iv—intravenous; T—thiamazole; P—prednisone; od—once a day; bd—twice a day; tds—three times a day; FT4—free T4; FT3—free T3; RR—reference range; MP—methylprednisolone.

On admission, he had episodes of confusion and hypotension (about 80/60 mmHg) associated with heart rates above 130/minute, BMI 22.6 kg/m^2^. He was markedly thyrotoxic, as shown by the following: FT4 > 7.77 ng/dL (0.93–1.7); FT3 14.9 pg/mL (2.0–4.4). His thyroid ultrasound was unremarkable, with unenhanced Doppler flow, while titres of all anti-thyroid antibodies were normal. He was treated with thiamazole 20 mg tds, prednisone 40 mg od, pulse MP 500 iv and then 250 mg iv once a week, as described by Campi et al. [7]. The course of his treatment is presented in Figure 4.

After six doses, his FT3 decreased to 2.05 pg/mL and his FT4 decreased to 2.41 ng/dL. Methylprednisolone was stopped. After about four weeks (despite the continuation of thiamazole 20 mg tds and oral prednisone 40 mg od), he presented with rebound thyrotoxicosis, as shown by the following: FT4 > 7.77 ng/dL; FT3 5.46 pg/mL. Methylprednisolone was restarted (500 mg and then five doses of 250 mg) and was followed by a marked decline in FT4 to 2.51 ng/dL and in FT3 to 1.92 pg/mL. He subsequently underwent a successful thyroidectomy; one week post-thyroidectomy, his results were as follows: FT4: 0.92 ng/dL; FT3: 1.3 pg/mL; TSH: 0.06 µIU/mL.

### 2.5. Case V

A 78-year-old man (BMI 27.72 kg/m^2^) was admitted to the Department of Endocrinology and Metabolic Diseases due to refractory thyrotoxicosis. The patient had a history of atrial fibrillation. In March 2021, he developed COVID-19 infection, without any need for assisted ventilation. He was, however, hospitalised in District Hospital and at that point he was diagnosed with concomitant thyrotoxicosis. Further history revealed that he lost about 20 kg within 3–4 months prior to hospitalisation in District Hospital. In view of palpable goitre, he was stated on thiamazole 20 mg bd, but without any improvement in his thyroid function tests, which prompted his referral to our Department. He initially denied taking any amiodarone, but was not certain of his medication prior to hospitalisation in District Hospital. In such circumstances (after admission to our Department), the search for his medication was performed by his family, who sent a photo of packages of his medications, and this revealed that indeed he had been taking Opacorden^®^, Polpharma, Poland, i.e., Amiodarone 200 mg od. He probably had been using the drug for over a year.

In addition, he had a history of relatively well-controlled type 2 diabetes, hypertension and chronic ischaemic heart disease. Computer tomography of the chest, performed during hospitalisation in District Hospital, showed that his thyroid gland was significantly enlarged, especially the left lobe reaching the aortic arch, causing the narrowing and right-sided displacement of the trachea, however without causing any stridor.

**Figure 5:**
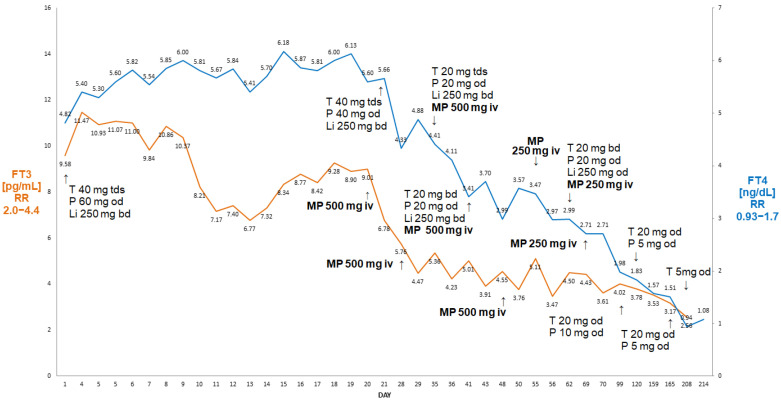
Summary of concentrations of free T4 and free T3 of a 78-year-old male patient with AIT. There was a decline in free T4 and free T3 following the initiation of weekly ivMP finally leading to cure without a need to resort to thyroidectomy. Abbreviations: iv—intravenous; T—thiamazole; P—prednisone; Li—lithium carbonicum; od—once a day; bd—twice a day; tds —three times a day; FT4—free T4; FT3—free T3; RR—reference range; MP—methylprednisolone.

On admission to our department, the patient was in a stable condition, with clinical and biochemical features of thyrotoxicosis (Figure 5).

The autoimmune disease of the thyroid was excluded (negative titres of ATPO, ATG and anti-TSH-receptor antibodies). Fine-needle aspiration of the dominant left-lobe lesion revealed benign changes, i.e., category II according to the Bethesda System. In view of atrial fibrillation with a ventricular rate of up to 150 beats per minute, a cardiological consultation was requested and an ECHO of the heart was performed. This did not reveal any significant structural cardiac abnormality and a dose of a beta blocker (nebivolol) was increased from 5 to 10 mg od. During admission to our department, he received treatment for thyrotoxicosis with high doses of intravenous thiamazole (120 mg/day), together with prednisone 60 mg od and lithium carbonate 250 mg bd. Initially, a downward trend in FT3 was observed (i.e., a decline from 11.5 pg/mL to 6.77 pg/mL), however with a subsequent rebound increase to 9.01 pg/mL, and no effect on FT4 concentrations (Figure 2). His condition was additionally complicated by the development of progressive anaemia (haemoglobin down to 7.6 g/dL) that required the transfusion of two units of red blood cells. Abdominal imaging studies failed to reveal any obvious malignancy, but his overall condition, in our opinion, rendered him unsuitable for emergency thyroidectomy.

Considering these circumstances, we decided to administer MP iv pulses (initially 500 mg iv, then 250 mg iv weekly) on the background of oral steroids (prednisone 60 mg od, then tapered to 40 and 20 mg od) as suggested by Campi at al. [6]. After six doses of ivMP, he became biochemically euthyroid, with a return of the sinus rhythm. His haemoglobin remained stable at about 11 g/dL. The patient is monitored as an outpatient, currently being off steroids and only on low-dose thiamazole (5 mg od). He was referred for a further examination of his anaemia (now stable), including elective colonoscopy and gastroscopy.

## 3. Discussion

Amiodarone-induced thyrotoxicosis may be extremely difficult to treat, while differentiation between type 1 and type 2 is not always feasible on clinical grounds. Glucocorticoids are considered to represent a treatment of choice for type 2 (destructive thyroiditis) AIT. Bogazzi et al. [8] used oral prednisolone (0.5 mg/kg of body weight) in 66 patients with type 2 AIT and achieved a cure in 60% within 30 days, and in about 85% within 90 days of treatment. Factors associated with good prognosis included a serum basal FT4 concentration no greater than 50 pg/mL (i.e., no more than about three times upper limit of normal) and a thyroid volume (normalised for body surface area) no greater than 12 mL/m^2^.

Though the use of MP in combination with methimazole was first described by Minelli et al. in 1992 [9], the data on the use of pulse MP are limited. According to Capellani et al. [10], pulsed MP seems to have similar efficacy to that of oral steroids in AIT, though this conclusion was based on a very small number of subjects (i.e., n = three for iv. MP versus n = nine for oral steroids), and no combination therapy (i.e., iv plus oral steroids) was used. Campi et al. [7] reported the successful application of pulsed MP (500 mg once every 3–7 days if FT4 was > four times above the upper reference limit and 250 mg once a week if FT4 was < four times above the upper reference limit, for 3–11 weeks) in type 2 AIT, when combined with background oral glucocorticoids.

Our first patient had extremely high concentrations of FT4, with a rebound increase following an original fall, despite undergoing over three weeks of treatment with a high dose of thionamides and prednisone with the addition of lithium carbonate. A single dose of iv MP (500 mg iv), with the concomitant use of oral glucocorticoids (prednisone 60 mg od), resulted in a marked decrease in FT3 (by 41%), i.e., almost to a reference range, prior to thyroid surgery.

A full normalisation of FT3 was also observed in cases of less severe thyrotoxicosis (Case II and III) and eventually in a complicated case, Case IV. A successful thyroidectomy was performed in all of these cases despite the low ejection fractions (particularly Cases II and IV; indeed, the patient described in Case II was awaiting a heart transplant).

The need for an emergency thyroidectomy is well described in cases of AIT [11] and is recommended in cases of deteriorating cardiac function [2]. Despite the risk of thyroid storm, surgery appears to be a reasonably safe option in comparison to no treatment or to a continuation of failing medical treatment [12]. Therapeutic plasma exchange may be also of benefit prior to a thyroidectomy [13], though failures of this approach were also described [14]. As the risk of thyroid storm remains real, in our opinion, all measures should be applied to obtain the best possible FT3 concentrations prior to surgery, particularly in the setting of a high FT4, which remains as a sort of reservoir of thyroid hormones.

In our first three cases, we achieved a quick normalisation (or near normalisation in Case I) of FT3 after relatively a small number of MP pulses (one in the first case, three in Case two and two pulses in Case three). It might be argued that in Case II we might have achieved normalisation even without the addition of ivMP (there was a simultaneous increase in the dose of thiamazole when intravenous MP treatment was started), but the addition of MP resulted in the normalisation of FT3 just after five days of treatment. This prevented yjr prolonged administration of high doses of thiamazole with the possible danger of a relapse of AIT on dose reduction as seen in Case I. Furthermore, cardiologists insisted on the possible reinstitution of amiodarone treatment following thyroidectomy in view of a considerable risk of ventricular dysrhythmias.

There are, however, caveats in treatment with MP, as demonstrated in Case IV. Thyroidectomy was not planned for this patient, and we achieved a normalisation of FT3 and the near normalisation of FT4 after six pulses of MP (three pulses of 500 mg and three pulses of 250 mg) in addition to oral steroids. While we expected an eventual favourable outcome, i.e., an eventual cure on medical therapy, there was a striking relapse of thyrotoxicosis with an increase in FT4 above the upper assay detection limit within four weeks after the last MP pulse, despite the continuation of treatment with high-dose thiamazole and oral prednisone. It might be argued that pulsed MP treatment was discontinued prematurely in this case; however, there are no clear literature data on the optimal duration of ivMP administration, particularly in the setting of FT3 concentrations close to the lower reference range, near the normalisation of FT4 with the continuing administration of oral prednisone. The second course of iv MP pulses (one 500 mg pulse and five 250 mg pulses) was successful, but the patient eventually was also referred for thyroidectomy. This contrasts with the situation described in Case V, where a prolonged course of iv. MP in addition to oral steroids led to a full remission without a need to resort to thyroidectomy, which might have been risky in this subject, an elderly subject with several co-morbidities, including atrial fibrillation, type 2 diabetes and unexplained anaemia. It should be noted that prior to the administration of iv. MP, the patient described in Case V received very high doses of thionamides together with high-dose prednisolone, as well as lithium carbonate without apparent biochemical improvement (and indeed there was a rebound increase in FT3).

It might also be argued that the continuation of thiamazole was probably unnecessary in view of the initial failure of this treatment, yet current guidelines, e.g., those of Ylli et al. [15], suggest a continuation of thiamazole prior to thyroidectomy in cases of refractory AIT.

In all our patients, there were no significant adverse effects of MP administration, apart from mild increases in ALT in Case IV (<100 U/L), that could also be associated with high-dose thionamides. The intravenous administration of MP (well investigated for the treatment of Graves’s ophthalmopathy) has been associated with liver dysfunction and about 0.6% mortality [16], but this was applicable to patients who exceeded a cumulative dose of MP of 8 g. Euguchi et al. [17] analysed a series of 175 Japanese patients with Graves’s ophthalmopathy treated with iv MP. Though slight increases in ALT (40–100 U/L) were common (35% of patients), only 10 patients (6%) had ALT within 100–300 U/L and only 7 patients (6%) had ALT > 300 U/L. The cumulative dose of MP was a dominant factor, though male sex and ages above 50 were also contributory to overall iv MP toxicity. Interestingly, however, all patients with ALT >300 U/L were female.

The highest cumulative doses of MP in our patients were 3.75 g (Case IV) and 3.25 g (Case V), respectively. It should be noted, however, that in Case Iv, thyroidectomy could have already been performed after six pulses of MP (2.25 g), i.e., before the discontinuation of MP treatment, which lead to rebound thyrotoxicosis.

Hence, in our opinion the risk of potential hepatic dysfunction after ivMP for AIT appears to be lower than that in Graves ophthamopathy, particularly if this therapy is used as an adjunct treatment in preparation for thyroidectomy, where the normalisation of FT3 can be achieved relatively quickly. Furthermore, the application of ivMP shortens the overall duration of medical therapy, while it has been recently demonstrated that unsuccessful medical therapy is one of the leading factors in increased mortality in cases of AIT [18]. In our patients, we also did not observe any significant hyperglycaemia, thought it must be taken into account that none of them had uncontrolled type 2 diabetes or a BMI > 30 kg/m^2^, while some of them lost a considerable amount of weight prior to admission, thus decreasing insulin resistance.

Another interesting issue pertains to the potential relationship between COVID-19 infection and the course of AIT in Case V. A review of the history (e.g., weight loss over the period of 3–4 months) clearly indicated that symptoms of AIT antedated COVID-19 infection. It is worth mentioning that COVID-19 infection was reported to cause transient thyroid dysfunction and/or to worsen pre-existing thyroid disease in about 15% of subjects [19], and potentially, it may also cause thyroiditis, including cases of subacute thyroiditis [20]. It remains, however, purely speculative, whether or not COVID-19 infection might have contributed to such a refractory course of AIT in our patient.

Our data are based on a similar number of patients as that described in the study by Campi et al. [7], i.e., five patients versus four. According to Campi et al. [7], the starting dose of each iv MP pulse was 250 or 500 mg, depending on the FT4 serum levels (<4 times the upper limit of the normal value  =  250 mg, >4 times the upper limit of the normal value  =  500 mg). We confirm the efficacy of ivMP in addition to oral steroids as an adjunct treatment of severe type 2 AIT. Campi et al. pointed out that 500 mg iv. MP corresponds to 625 mg of prednisone. Moreover, MP has a higher anti-inflammatory effect than prednisone does [21], so this might be a mechanism responsible for the success of such an approach. Furthermore, the data derived from the treatment of Graves’s ophthalmopathy [22] indicate that ivMP has less side effects than do escalating doses of oral steroids, and appears reasonably safe as long as the cumulative dose of eight grams is not surpassed. This also includes a relatively low risk of iatrogenic adrenal insufficiency [23].

## 4. Conclusions

Pulse intravenous methylprednisolone in addition to oral steroids may be a useful adjunct therapy either for the preparation for thyroidectomy, or in restoring euthyroidism in cases of drug-resistant amiodarone-induced thyrotoxicosis. Caution, however, is needed as the interruption of pulse methylprednisolone treatment may lead to a relapse of thyrotoxicosis despite the continuation of thiamazole and oral glucocorticoids.

## Data Availability

Data are contained within the article.
